# Giant Orbital Anisotropy with Strong Spin–Orbit Coupling Established at the Pseudomorphic Interface of the Co/Pd Superlattice

**DOI:** 10.1002/advs.202201749

**Published:** 2022-06-24

**Authors:** Sanghoon Kim, Sachin Pathak, Sonny H. Rhim, Jongin Cha, Soyoung Jekal, Soon Cheol Hong, Hyun Hwi Lee, Sung‐Hun Park, Han‐Koo Lee, Jae‐Hoon Park, Soogil Lee, Hans‐Georg Steinrück, Apurva Mehta, Shan X. Wang, Jongill Hong

**Affiliations:** ^1^ Department of Materials Science and Engineering Yonsei University Seoul 03722 Korea; ^2^ Department of Physics University of Ulsan Ulsan 44610 Korea; ^3^ Pohang Acceleration Laboratory Pohang 37673 Korea; ^4^ Department of Physics Pohang University of Science and Technology Pohang 37673 Korea; ^5^ Department of Chemistry Paderborn University Paderborn 33098 Germany; ^6^ SSRL Materials Science Division SLAC National Accelerator Laboratory CA 94025 USA; ^7^ Department of Materials Science and Engineering and Electrical Engineering Stanford University CA 94305 USA; ^8^ Present address: Department of Physics University of Ulsan Ulsan 44610 Korea

**Keywords:** anisotropic orbital structures, Berry phase, pseudomorphic interface, spin–orbit coupling

## Abstract

Orbital anisotropy at interfaces in magnetic heterostructures has been key to pioneering spin–orbit‐related phenomena. However, modulating the interface's electronic structure to make it abnormally asymmetric has been challenging because of lack of appropriate methods. Here, the authors report that low‐energy proton irradiation achieves a strong level of inversion asymmetry and unusual strain at interfaces in [Co/Pd] superlattices through nondestructive, selective removal of oxygen from Co_3_O_4_/Pd superlattices during irradiation. Structural investigations corroborate that progressive reduction of Co_3_O_4_ into Co establishes pseudomorphic growth with sharp interfaces and atypically large tensile stress. The normal component of orbital to spin magnetic moment at the interface is the largest among those observed in layered Co systems, which is associated with giant orbital anisotropy theoretically confirmed, and resulting very large interfacial magnetic anisotropy is observed. All results attribute not only to giant orbital anisotropy but to enhanced interfacial spin–orbit coupling owing to the pseudomorphic nature at the interface. They are strongly supported by the observation of reversal of polarity of temperature‐dependent Anomalous Hall signal, a signature of Berry phase. This work suggests that establishing both giant orbital anisotropy and strong spin–orbit coupling at the interface is key to exploring spintronic devices with new functionalities.

## Graphical abstract

The unique pseudomorphic interfacial structure of Co/Pd constructed by phase‐transformation of paramagnetic Co_3_O_4_/Pd to ferromagnetic Co/Pd superlattices by low‐energy proton irradiation makes it possible to induce extraordinarily large orbital anisotropy and strongly enhances spin–orbit coupling (SOC) at the interface, which can be attributed to the strong level of structural inversion asymmetry (SIA) and the substantial in‐plane tensile stress built up at the interface between Co and Pd.

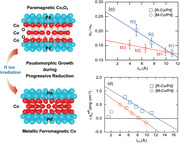

## 1 Introduction

Heterostructures composed of ultrathin layers of heavy metal (HM) and ferromagnetic metal (FM) have shown newly emerging spin–orbit related phenomena, such as the Rashba effect,^[^ ^1^, ^2^, ^3^ ^]^ spin–orbit torque,^[^ ^4^, ^5^ ^]^ and Dzyaloshinskii–Moriya interaction (DMI).^[^ ^6^, ^7^, ^8^, ^9^, ^10^ ^]^ These phenomena all share common features that originate from two essential physical effects: structural inversion asymmetry (SIA) and spin–orbit coupling (SOC). These HM/FM heterostructures enable the control of magnetization by electrical current^[^ ^4^, ^5^, ^11^, ^12^ ^]^ and the creation of chiral spin textures such as magnetic skyrmions.^[^ ^13^, ^14^, ^15^ ^]^

Recent intriguing observations associated with SOC^[^ ^16^, ^17^ ^]^ in HM/FM heterostructures reveal that the orbital angular momentum is no longer fully quenched at the interface but exhibits substantial asymmetric magnitude due to hybridization at the interface. This anisotropic orbital hybridization at the interface is starting to receive significant attention since it can provide physical clues to the aforementioned spin–orbit‐related phenomena.^[^ ^18^, ^19^, ^20^ ^]^ In spite of extensive experiments with various methods to modulate the interfacial hybridization, such as *δ*‐layer insertion at the interface or thermal treatment in the HM/FM heterostructures,^[^ ^21^, ^22^ ^]^ an efficient method has not yet been discovered. In this context, an attempt to control orbital anisotropy at the atomic scale is indispensable for further exploring spin–orbit‐related phenomena.

Here, we report that the phase‐transformation from Co_3_O_4_/Pd to Co/Pd superlattices by low‐energy proton irradiation^[^ ^23^, ^24^ ^]^ induces extraordinarily large orbital anisotropy and strongly enhances SOC at the interface, which can be attributed to the strong level of SIA and the substantial in‐plane tensile stress built up at the interface between Co and Pd. The contribution of orbital to spin magnetic moments in the Co/Pd superlattice along the interface normal is as exceptionally large as that found in the Co atomic chains.^[^ ^25^ ^]^ An observation of polarity change in the anomalous Hall resistivity arising from the Berry phase can provide evidence for the giant orbital anisotropy with strong SOC in the Co/Pd superlattice. The unique pseudomorphic interfacial structure of Co/Pd is key to the observation of those intriguing attributes. Our work lends support to the prediction that to discover newly emerging spin–orbit related phenomena, the necessary and sufficient condition is the presence of both strong SOC and giant orbital anisotropy.

## 2 Results

### 2.1 Experimetal Analysis of Magnetic Moments

**Figure 1:**
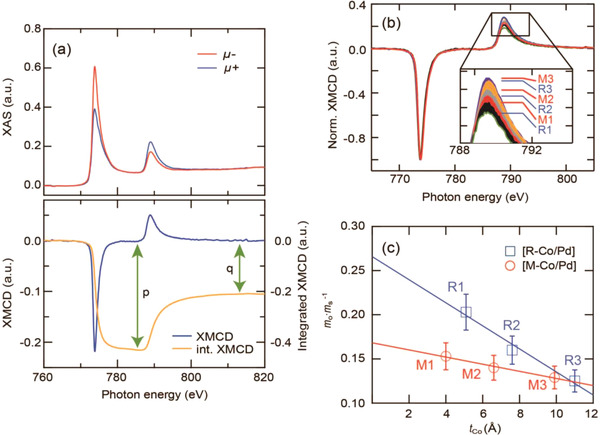
a) XAS, XMCD, and integrated XMCD *L*_2,3_ spectra of Co in the [R‐Co/Pd] at *t*_Co_ = 7.5 Å. The red and blue lines are XAS spectra measured when magnetizations of the superlattice were antiparallel (*μ*_−_) and parallel (*μ*_+_), respectively, to the incident direction of the 95% circularly polarized X‐ray. b) XMCD spectra normalized by the Co *L*_3_ edge intensities. The [R‐Co/Pd] with *t*_Co_ of 5, 7.5, and 11 Å are labeled R1, R2, and R3, respectively, and the [M‐Co/Pd] with *t*_Co_ of 4, 6.5, and 10 Å are labeled M1, M2, and M3, respectively. c) The ratio of orbital‐to‐spin magnetic moment (*m*_o_/*m*_s_) as a function of *t*_Co_.

An exceptionally large contribution of interfacial orbital moments to the total magnetic moment in the [reduced Co 5/Pd 10 (Å)]_10_ superlattice (denoted [R‐Co/Pd] hereafter) was confirmed by X‐ray magnetic circular dichroism (XMCD) spectroscopy. The thickness of reduced Co stays 5 Å throughout this article unless otherwise specified. The [R‐Co/Pd] is a metallic ferromagnet that was phase‐transformed from paramagnetic [Co_3_O_4_/Pd]_10_ through selective removal of oxygen (or reduction) in the Co oxide by low‐energy proton irradiation.^[^ ^23^, ^24^ ^]^ **Figure** **1**a shows the X‐ray absorption (XAS), XMCD, and integrated XMCD spectra of the [R‐Co/Pd] at a reduced Co thickness of 0.75 nm (R2). The metallic [Co/Pd]_10_ superlattices with a layer structure identical to [R‐Co/Pd] were separately prepared as a reference (we denote it [M‐Co/Pd]). The X‐ray incident angle was perpendicular to the film plane during measurements. Therefore, what we present in this article is closely related to the physical properties along the film normal. The ratio of orbital‐to‐spin magnetic moment *m*_o_ */m*_s_ is estimated based on the sum rule using the difference between the integrated intensities of *L*_3_ and *L*_2_ edges: *m*_o_ */m*_s_ = *2q/(9p−6q)*,^[^ ^16^, ^25^, ^26^, ^27^, ^28^ ^]^ where *p* and *q* are integral values of the XMCD spectra denoted in Figure 1a (see more details in the Supporting Information). The orbital moment contribution is proportional to the *q* value that corresponds to the difference between the absolute integral values of the *L*_3_ and *L*_2_ edge. Figure 1b shows the integrated XMCD spectra of the [R‐Co/Pd] as a function of Co thickness. Since the spectra are normalized by the intensity at the *L*_3_ edge, the relative strength of the *L*_2_ intensity (or equivalently the ratio of *m_o_*/*m*_s_) directly indicates the degree of the orbital contribution in the superlattice. Our measurements clearly show that the *L*_2_ peak is strongly dependent on the thickness of Co in both superlattices; i.e., thinner Co results in smaller *L*_2_ intensity. This suggests an important implication: the orbital magnetic moment arising from the interfaces is not fully quenched. The calculated value of *m*_o_ */m*_s_ along the film normal is shown in Figure 1c. The ratio for both superlattices linearly increases with decreasing Co thickness. The interfacial orbital contribution at the interface can be estimated by a linear curve‐fit in the plot of *m*_o_/*m*_s_ versus *t*_Co_ *_,_* and it corresponds to the *y*‐intercept of the fit. Note that the interfacial orbital contribution is larger of the [R‐Co/Pd] than that of the [M‐Co/Pd] by ≈60%: 0.27 versus 0.17. The orbital contribution of the [R‐Co/Pd] is even larger at room temperature than that of a trilayer of Au/Co/Au (≈0.22).^[^ ^16^ ^]^ To the best of our knowledge, our *m*_o_ */m*_s_ of the [R‐Co/Pd] is the largest value among those ever reported in 2D or layered Co systems.^[^ ^16^, ^25^, ^26^, ^27^, ^28^ ^]^

### 2.2 Characteristics of the Pseudomorphic Interface

**Figure 2:**
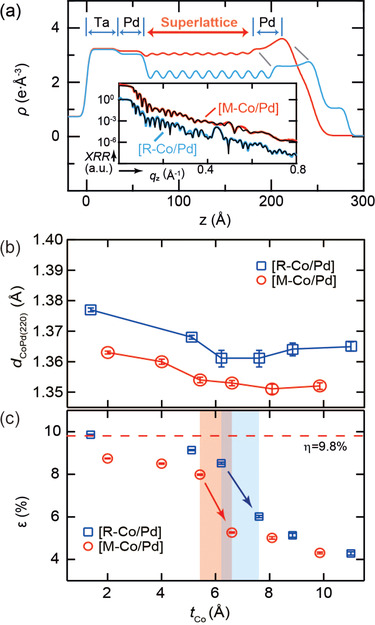
a) X‐ray reflectivity‐derived electron density (*ρ*) profile of [R‐Co/Pd] and [M‐Co/Pd] as a function of film depth (*z*). The inset shows the X‐ray reflectivity of the superlattices along with the model fits. b) Changes in the *d*‐spacing of the Co/Pd (220) plane in the [R‐Co/Pd] and the [M‐Co/Pd] with *t*_Co_ measured by GIXRD. c) Changes in the in‐plane strain (*ε*) in the [R‐Co/Pd] and the [M‐Co/Pd] with *t*_Co_. The red dotted line indicates the lattice mismatch *η* of 9.8% between Co and Pd. The shaded blue and reddish regions indicate the thickness range in which strain relaxation starts to occur in the [R‐Co/Pd] and the [M‐Co/Pd], respectively.

Our investigations into structures of the [R‐Co/Pd] by X‐ray reflectivity (XRR) and grazing incident X‐ray diffraction (GIXRD) provide clear answers to the question of how such enhanced interfacial orbital contribution can be achieved. First of all, XRR spectra confirm the existence of a well‐defined clean (i.e., abrupt) interface in the case of [R‐Co/Pd]. **Figure** **2**a shows the electron density profiles (EDPs) along the depth (*z*) obtained from XRR spectra (inset) for the two superlattices. (The inset shows the XRR data together with the corresponding model fits.) The EDP for *z* < 0 corresponds to the silicon substrate; the EDP for 0 < *z* ≲ 50 and 200 ≲ *z* ≲ 250 correspond to the Ta and Pd seed layers and the Pd protection layer; the oscillatory part of the EDP for 50 < *z* ≲ 200 corresponds to the superlattice. The oscillation originates from the difference in electron density between Co and Pd, a manifestation of sharp interfaces. Notably, the amplitudes of oscillations are significantly larger in the [R‐Co/Pd]. This is an indication of a sharp transition between individual layers, which is also observed in the largest amplitude of the peak at around 0.45 Å^−1^ in the XRR. For the [M‐Co/Pd], the oscillations appear but are less pronounced due to the intermixing of Co and Pd atoms at the interface. This implies that the interface of [R‐Co/Pd] is more discrete than that of [M‐Co/Pd]. In other words, the interface of [R‐Co/Pd] exhibits a stronger level of SIA than that of [M‐Co/Pd] does, which results in strong Néel surface anisotropy and anisotropic orbital hybridization at the interface between Co and Pd (more of which later). The SIA and atomic SOC at the interface have been known to greatly influence orbital hybridization.

Next, we also investigate the strain state of Co/Pd superlattices since orbital hybridization at interfaces is strongly dependent on the degree of lattice strain in the superlattice.^[^ ^22^, ^23^, ^24^, ^25^, ^26^, ^27^, ^28^, ^29^ ^]^ In order to quantify the lattice strain, the *d*‐spacing of the Co/Pd (220), whose crystallographic direction is parallel to the in‐plane direction, is analyzed by GIXRD (Supporting Information). Both superlattices are confirmed to have preferred growth orientation along the [111], which is perpendicular to the (220). In general, because of stress relaxation during the deposition of Co/Pd superlattices, the interatomic distance between Pd atoms in the plane decreases while that between Co atoms increases, which is shown in the case of [M‐Co/Pd]. Note that the Co/Pd (220) *d*‐spacing of [R‐Co/Pd] is larger than that of [M‐Co/Pd], as shown in Figure 2b. Stress is also relaxed in the case of [R‐Co/Pd]. However, the Co/Pd (220) *d*‐spacing in the plane is quite different from that found in the [M‐Co/Pd]. The Co/Pd (220) *d*‐spacing in the plane is similar to that of bulky Pd (1.37 Å) when *t*_Co_ = 5 Å.

The dependence of *t*_Co_ on the in‐plane strain of Co layers (*ε*_Co_) in Co/Pd superlattices, as shown in Figure 2c, provides important evidence for the origin of giant orbital anisotropy. Unlike variations of the Co/Pd (220) *d*‐spacing, the strain of Co layers calculated based on the compositionally volume‐weighted average *d*‐spacing reveals an abrupt decrease in the strain at *t*_C_ = ≈6 Å for both superlattices, as indicated by the red and blue arrows (see analyses in the Supporting Information). This is due to a sudden release of stress in the superlattice, which is usually observable in the pseudomorphic growth mode. Co tends to grow on Pd layer‐by‐layer, followed by an island mode due to the surface energy of Pd being lower than that of Co (1600 vs 2000 erg cm^−2^) in Co/Pd superlattices, which is known as the Stranski–Krastonov model.^[^ ^30^, ^31^ ^]^ When *t*_Co_ < *t*_C_, Co is expected to grow on Pd pseudomorphically layer‐by‐layer under tensile stress. On the other hand, when *t*_Co_ > *t*_C_, Co can form an island as a result of stress relaxation. Here, *t*_C_ is the critical thickness at which the coherent strain begins to relax. Thus, our observation of the *t*_Co_ dependence clearly supports the pseudomorphic growth of both Co/Pd superlattices when *t*_Co_ < *t*_C_. However, caution should be taken here. Although both Co/Pd superlattices are pseudomorphically grown, their interfacial states are quite different from each other in terms of sharpness and strain at the interface and inter‐atomic distance of ferromagnetic Co. For example, the [R‐Co/Pd] has sustained strain over 9% even though the interatomic distance of Co in the plane is stretched to that of bulky Pd when *t*_Co_ = *t*_C_. Therefore, the unique pseudomorphic nature in our [R‐Co/Pd], which is challenging to create by conventional methods, can have a significant influence on the orbital hybridization at the atomic scale at the interface.

**Figure 3:**
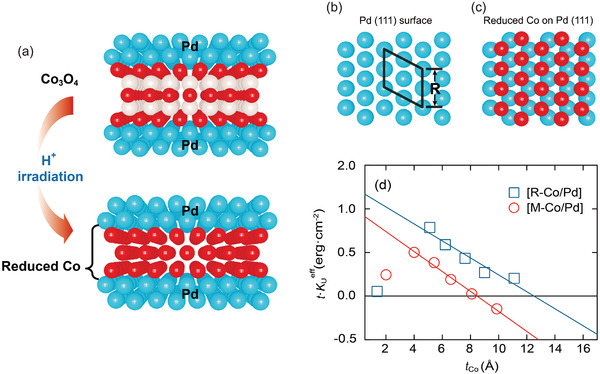
a) Schematics of the interface in the Co_3_O_4_/Pd superlattice and [R‐Co/Pd]. The white, red, and blue spheres denote O, Co, and Pd atoms, respectively. b) Schematic plan‐view of the Pd (111) surface and c) schematic plan‐view of the reduced Co atoms on the Pd (111) surface. *R* indicates the side length of a 2×2 surface unit mesh in the (111) plane. d) Changes in *K*_u_^eff^ with *t*_Co_ for the [M‐Co/Pd] and the [R‐Co/Pd]. The red and blue solid lines are the fitted lines.

Our pseudomorphic growth of [R‐Co/Pd] with these unique interfacial states is the result of peculiar growth, in which a progressive reduction process occurs during the proton irradiation, as illustrated in **Figure** **3**a. Here, we use a (2×2) surface unit mesh with a side length *R* in the (111) plane (see Figure 3b). When the [Co_3_O_4_/Pd] superlattice is initially prepared, the metallic (111) Pd deposited “on top of Co_3_O_4_” should experience tensile stress because the *R* of Co_3_O_4_ is larger than that of Pd (5.71 vs 5.50 Å). As a result, Pd in the [Co_3_O_4_/Pd] superlattice has a lattice parameter larger than bulk Pd has. In addition, the interface can be well defined due to weak interdiffusion between metal (Pd) and oxide (Co_3_O_4_). After the [Co_3_O_4_/Pd] is prepared, protons are irradiated to reduce oxide in order to create the [R‐Co/Pd]. When oxygen atoms are selectively removed by the momentum transfer from the energetic protons, the leftover Co atoms can fit into the interstices in the Pd layer, which results in the pseudomorphic growth of Co on just a 10 Å‐thick Pd seed‐layer, as illustrated in Figure 3c. At the same time, Pd atoms are not significantly relaxed from the tensile stress created in the as‐prepared state due to hydrogen implantation. It has been reported that there can be a ≈2% volume expansion of Pd when hydrogenation takes place.^[^ ^32^ ^]^ This may indicate that the hydrogen atoms inside the [R‐Co/Pd] can hamper the relaxation during the reduction process without affecting the orbital hybridization (see Supporting Information). As a consequence, Co atoms settled on the Pd layer are under extraordinarily large stress: the lattice mismatch *η* being about 9%. In the case of [M‐Co/Pd] or most of the Co/Pd superlattices, Pd atoms should experience compressive stress due to the lattice mismatch to reduce the elastic energy^[^ ^33^ ^]^ simply because the lattice parameter of Co is much smaller than that of Pd.

### 2.3 Interfacial Magnetic Anisotropy

Two important effects, the large interfacial orbital contribution and the unusually strained pseudomorphic interface, should have an impact on the magnetic characteristics of the [R‐Co/Pd]. We find that the interfacial magnetic anisotropy is increased by ≈30% over that in the [M‐Co/Pd], as shown in Figure 3d and summarized in **Table** **1**. Figure 3d shows a plot of *K*_u_^eff^∙*t*_Co_ versus *t*_Co_, where *K*_u_^eff^ is the effective magnetic anisotropy estimated by the areal method (see details in the Supporting Information).^[^ ^34^ ^]^ *K*_u_^eff^ can phenomenologically be decomposed into *2K*_s_ */t*_Co_ and *K*_v_, where *K*_s_ is the anisotropy of the interface per unit area and *K*_v_ is the volume anisotropy of Co. More specifically, *K*_s_ taken from the *y*‐intercept in Figure 3d includes the Néel anisotropy (*K*_N_) and the strain‐induced anisotropy (*K*_s_^inc^) due to incoherent strain. *K*_N_ and *K*_s_^inc^ are attributed to SIA and the strain at the interfaces, respectively. Here, the *K*_s_^inc^ is defined as *K*_s_^inc^ = 3/4*λE*_Co_ *ηt*_C_,^[^ ^34^ ^]^ where *λ* and *E*_Co_ are the magnetostriction coefficient and the Young's modulus of Co, respectively.^[^ ^35^ ^]^ On the other hand, the Néel surface anisotropy can be calculated as *K*_N_ = *K*_s_ − *K*_s_^inc^. The interfacial contributions in the [R‐Co/Pd], *K*_s_^inc^ and *K*_N_, are significantly enhanced by 37% and 18%, respectively, over those in the [M‐Co/Pd] (see Table 1). This clearly indicates that the unique pseudomorphic nature of [R‐Co/Pd] has dramatically enhanced not only the magneto‐elasticity but also the level of SIA at the interface.

**Table 1:** Interface and volume anisotropy contributions estimated from the *K*_u_^eff^∙*t*_Co_ versus *t*_Co_ plot in Figure 3

	*K*_s_^inc^ [erg cm^−2^]	*K*_N_ [erg cm^−2^]	*K*_s_ [erg cm^−2^]	*K_v_* [erg cm^−3^]
[R‐Co/Pd]	0.22	0.39	0.61	−1.0 × 10^7^
[M‐Co/Pd]	0.16	0.33	0.49	−1.1 × 10^7^

### 2.4 Theoretical Calculations

**Figure 4:**
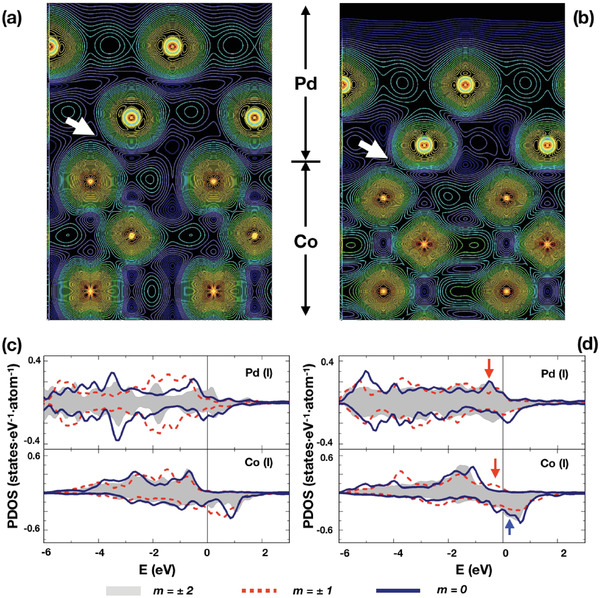
Charge density and partial DOS of d orbitals of the unstrained Co/Pd (Co lattice constant) and the strained Co/Pd with 10% strain (Pd lattice constant). A charge of an energy window of *E*_F_ ± 0.1eV is chosen for a) the unstrained Co/Pd and b) the strained Co/Pd. Partial density of d orbitals of interface atoms (denoted by I): *m* = ±2 (shaded in grey, *m* = ±1 (red dashed line), and *m* = 0 (solid line), respectively, for c) the unstrained Co/Pd and d) the strained Co/Pd. Fermi level is set to zero.

The strong anisotropy of the interfacial orbitals is further confirmed by first‐principles calculations (see details in the Supporting Information). We have chosen two extreme systems—unstrained Co/Pd and strained Co/Pd with 10% strain—for the calculation, to provide a large contrast. The strained Co/Pd simulates the [R‐Co/Pd]. **Figure** **4**a,b shows the iso‐charge density plot of the two Co/Pd, where charges in an energy window of ±0.1 eV relative to the Fermi level (*E*_F_) are taken into account. The vertical axis is along the [001] whereas the horizonal one is along the [120] connecting two hexagonal sites. The iso‐density plot is dominated by Co sites, as states near *E*_F_ are mainly of Co d orbitals. Evidently, the unstrained and the strained Co/Pd superlattices exhibit different interfacial charges. As indicated by the white arrows, the interfacial charge in the strained Co/Pd is distributed broader than it is distributed in the unstrained Co/Pd. We further analyze the interfacial charges and estimate the magnitude of orbital moments based on Bruno's relation:^[^ ^36^ ^]^ *E*_MCA_ = *V*_SOC_(*m*_o_^┴^−*m*_o_ *^║^*) where *E*_MCA_ and *V*_SOC_ are the magneto‐crystalline anisotropy (MCA) energy and the strength of SOC, respectively, and *m*_o_^┴^ and *m*_o_^║^ are the orbital moments when magnetization is perpendicular to the plane and is in the plane, respectively. For simplicity and without loss of generality, *E*_MCA_ is mainly determined by a change in the orbital magnetic moments, Δ*m*_o_ = *m*_o_^┴^−*m*_o_^║^. Δ*m*_o_ serves as a direct indicator of the orbital anisotropy. It turns out that Δ*m*_o_ is 0.032 *μ*_B_ for the strained Co/Pd and Δ*m*_o_ is 0.008 *μ*_B_ for the unstrained Co/Pd, an increase in Δ*m*_o_ by 400% over the unstrained Co/Pd, which indicates that the strained Co/Pd indeed has giant orbital anisotropy (see details in the Supporting Information).

Figure 4c,d shows the partial density of states (PDOS) of d orbitals of the unstrained Co/Pd and the strained Co/Pd, respectively. Since the (111) surface of a face‐centered cubic (FCC) lattice is hexagonal, the PDOS of *d_x_* ^2^ *_−y_* ^2^_/_ *_xy_*, *d_xz_*_/_ *_yz_*, and *d_z_* ^2^ orbitals of interface atoms are presented, as these are the irreducible orbitals according to group theory, with the magnetic quantum numbers of *m* = ±2, ±1, and 0, respectively. The PDOS of atoms away from the interface are shown in the Supporting Information for reference. MCA is discussed in the context of anisotropy and MCA itself following a second‐order perturbation scheme.^[^ ^37^ ^]^ The difference between the two superlattices is noticed near *E*_F_ in the occupied *m* = ±1 states of the majority spin channel and the empty *m* = 0 state in the minority spin channel. Those states in the strained Co/Pd shift toward *E*_F_ as denoted by the arrows in Figure 4d. As a consequence, PMA is enhanced by ⟨*m* = ±1,↑*|L_x_* |*m *= 0,↓⟩ owing to the dominant hybridization between *m* = ±1,↑ and *m* = 0,↓ states.

To obtain more insight, Co films without Pd are also investigated for comparison, where ⟨*m* = ±1,↑|*L_x_* |*m *= 0,↓⟩ contributes to *E*_MCA_ < 0. In the presence of Pd, this matrix is balanced by ⟨*m* = ±1,↑|*L_x_* |*m *= 0,↓⟩ which contributes to *E*_MCA_ > 0. Furthermore, as mentioned earlier, ⟨*m* = ±1,↑|*L_x_|m *= 0,↓⟩ enhances *E*_MCA_ > 0 much more in the strained Co/Pd than in the unstrained Co/Pd. This fact evidences that the existence of Pd at the interface along with strain is a crucial factor in obtaining such large orbital anisotropy in the [R‐Co/Pd]. Moreover, the relevant *m* = ±1 orbitals are strongly associated with orbital anisotropy, as observed in the experiment. We also confirm that the hydrogenation does not significantly affect the orbital structure in the Co/Pd, as shown in Supporting Information.

### 2.5 Observation of the Reversal of a Polarity of Anomalous Hall Signals as a Function of Temperature

**Figure 5:**
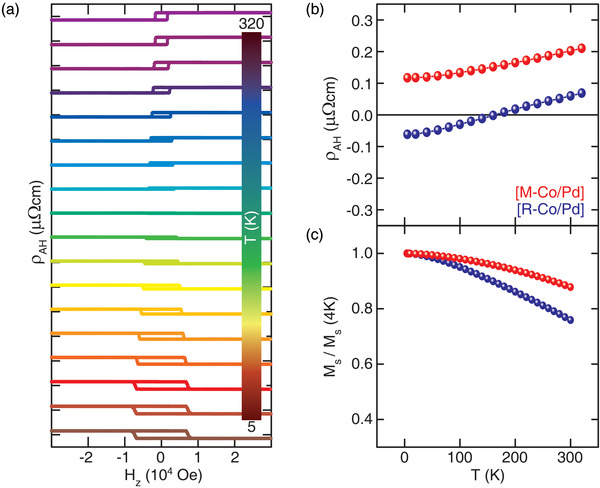
a) Anomalous Hall resistivity (*ρ*_AH_) as a function of *H_z_* for the [R‐Co/Pd] heterostructures with *H_z_* perpendicular to the film plane and the measurement for temperature range from 5 to 320 K. b) Temperature dependence of *ρ*_AH_ and c) normalized magnetization *M*_s_(*T*)/*M*_s_(4 K) for both [M‐Co/Pd] and [R‐Co/Pd].

The changes in the interfacial orbital state and the interfacial SOC should have an impact on the transport characteristics, such as AHE. In fact, the superlattice with the enhanced SOC, i.e., the [R‐Co/Pd], explicitly shows the reversal of the AHE loop's polarity as a function of temperature. **Figure** **5**a shows the anomalous Hall resistivity (*ρ*_AH_) loops measured at a temperature range from 5 to 320 K, with the external magnetic field along the *z*‐direction (*H_z_*), perpendicular to the film plane. A sharp switching is observed, which reflects strong PMA. The *ρ*_AH_ of the [R‐Co/Pd] changes from negative to positive at a temperature of 140 K, whereas that of the [M‐Co/Pd] shows only positive values and increases with increasing temperature, as shown in Figure 5b. The change in polarity of the [M‐Co/Pd] is absent in the temperature range. Unlike those conflicting behaviors, the magnetization of both superlattices monotonically decreases with increasing temperature (Figure 5c). Understanding these behaviors of *ρ*_AH_ is rather challenging since *ρ*_AH_ is a result of competition among intrinsic, side‐jump and skew scattering^[^ ^38^ ^]^ in addition to the complex scattering in a multilayered structure. In the case of [R‐Co/Pd], assuming that the extrinsic scattering is suppressed at low temperatures, the negative value of *ρ*_AH_ can be attributed to the scattering‐independent intrinsic contribution, i.e., to the Berry phase.^[^ ^39^ ^]^ Since strong SOC can open small bandgaps, and giant orbital anisotropy can significantly modulate the electron filling near the Fermi level, electrons experience nonvanishing Berry curvature. When temperature increases, the *ρ*_AH_ reverses its sign to positive values due to the temperature‐dependent extrinsic contribution: phonon and magnon scatterings increase both *ρ*_AH_ and *ρ_xx_* with temperature, which is a typical metallic behavior in electrical transport. Both [R‐Co/Pd] and [M‐Co/Pd] show a positive temperature coefficient of resistivity (TCR, d*ρ_xx_*/d*T*).

## 3 Conclusion

We found that our unique low‐energy proton irradiation method has established the pseudomorphic rearrangement of Co atoms reduced from the oxide phase, featuring a stronger level of SIA withstanding large tensile stress caused by the abrupt pseudomorphic interface between Co and Pd. As a result, the [R‐Co/Pd] shows giant orbital anisotropy and strongly enhanced SOC at the interface. In addition to the large PMA, the reversal of AHE polarity arising from the Berry phase supports the unique interfacial structure modified by our method. Our work demonstrates that a strongly asymmetric interface with unusual strain is a crucial requisite in pioneering new spin–orbit coupled phenomena in heterostructures and exploring new observations in systems where interfacial structures dominate.

## 4 Experimental Section

### Materials Synthesis and Device Fabrication

The oxide superlattices composed of thermally oxidized Si substrate/Ta 40/Pd 30/[Co_3_O_4_ *t*/ Pd 10]_10_/Pd 20 [Å] were prepared using an ultrahigh‐vacuum d.c. magnetron sputterer with a base pressure below 3.0×10^−9^ Torr. The size of the Si substrate was taken 8 mm×8 mm. The Co layers were reactively oxidized to Co_3_O_4_ during the sputter deposition. To reduce oxidic Co_3_O_4_ to metallic Co, proton plasma was generated with microwave power at 2.5 GHz using a microwave source. The protons were then extracted and accelerated through grids toward the superlattice at an acceleration voltage of 0.3–0.5 kV with a dose of more than 3.57×10^21^ ions m^−2^ in vacuum. Irradiated films were then patterned into a Hall‐bar structure (width (*w*) = 90 µm and length (*L*) = 5 µm) using electron beam lithography (EBL) for Hall measurements.

### Materials Characterizations of Deposited Films

The Co_3_O_4_ phase was confirmed by XRD using Cu K*α* radiation (*λ* = 0.154 nm) and X‐ray photoelectron spectroscopy measurements (not shown). XRR^[^ ^40^, ^41^ ^]^ measurements were performed at beamline 2‐1, Stanford Synchrotron Radiation Light (SSRL) Source, using 12 KeV X‐rays. XRR curves were fit using GenX software.^[^ ^42^ ^]^ GIXRD and XMCD spectra were obtained using the synchrotron source at the 5 and 2A beamlines of the Pohang Accelerator Laboratory (PAL), respectively. Taking into account experimental and analytic uncertainties errors, ± 10% error bars were conservatively applied to Figure 1c.^[^ ^43^ ^]^

### Electrical Measurements of the Device

The electrical characterizations of the patterned films with a Hall bar structure was carried out using a custom build system which consists of a Lake Shore electromagnet, Keithley 2400, and 2182 nano‐voltmeter. The anomalous Hall measurement is used to determine the longitudinal ( *ρ*_*xx*_ = (*V_x_*/*I_x_*)(*w*/*L*)*t* ) and transverse resistivity ( *ρ*_AH_ = (*V*_H_/*I_x_*) *t*) of the Hall‐bar device. Where, *t* is the thickness of films. Voltages (*V_x_* and *V*_H_) as a function of the applied magnetic field were measured to compute the resistivities (*ρ_xx_* and *ρ*_AH_) of the device. Data processing and analysis are carried out using Igor Pro software.

### Computational Modelling

First‐principles density functional theory calculations were carried out using the Vienna ab‐initio simulation program (VASP) and the generalized gradient approximation (GGA) in the form of Perdew–Burke–Ernzerhof (PBE) for the exchange‐correlation potential. The structures of Co/Pd and Co thin films were optimized using the k‐meshes of 18×18×2 and 18×18×1, respectively in Brillouin‐zone. The energy of magneto‐crystalline anisotropy is defined as the difference between total energies in‐plane and out‐of‐plane magnetization directions.

## Conflict of Interest

The authors declare no conflict of interest.

## Supporting information

Supporting Information

## Data Availability

The data that support the findings of this study are available from the corresponding author upon reasonable request.
