# Supplementary material for: Giant Orbital Anisotropy with Strong Spin–Orbit Coupling Established at the Pseudomorphic Interface of the Co/Pd Superlattice
Source: Adv Sci (Weinh). 2022 Jun 24;9(24):2201749. doi: 10.1002/advs.202201749 (PMC9403640; doi:10.1002/advs.202201749)
Supplement: Supplementary file 1 — Supporting Information [file ADVS-9-2201749-s001.pdf]

Supplementary Information

Giant Orbital Anisotropy with Strong Spin-Orbit Coupling Established at the Pseudomorphic Interface of the Co/Pd Superlattice

Sanghoon Kim,[1†] Sachin Pathak,[1†] Sonny H. Rhim,[2†] Jongin Cha,[1] Soyoung Jekal,[2] Soon Cheol Hong,[2] Hyun Hwi Lee,[3] Sung-Hun Park,[4] Han-Koo Lee,[3] Jae-Hoon Park,[4] Soogil Lee,[1] Hans-Georg Steinrück,[5] Apurva Mehta,[6] Shan X. Wang,[7] and Jongill Hong[1]*

Dr. S. Kim, Dr. S. Pathak, J. Cha, Dr. S. Lee, Prof. J. Hong
[1]Department of Materials Science and Engineering, Yonsei University, Seoul 03722, Korea.

Prof. S. H. Rhim, S. Jekal, Prof. S. C. Hong
[2]Department of Physics, University of Ulsan, Ulsan 44610, Korea.

Dr. H. H. Lee, Dr. H. Lee
[3]Pohang Acceleration Laboratory, Pohang 37673, Korea.

Dr. S. Park, Prof. J. Park
[4]Department of Physics, Pohang University of Science and Technology, Pohang 37673, Korea.

Dr. H. Steinrück
[5]Department of Chemistry, Paderborn University, Paderborn 33098, Germany

Dr. A. Mehta
[6]SSRL Materials Science Division, SLAC National Accelerator Laboratory, CA 94025, USA.

Prof. S. X. Wang
[7]Department of Materials Science and Engineering, and Electrical Engineering, Stanford University, CA 94305, USA.

[†]Authors equally contributed to this work

Present Address:
Sanghoon Kim[1†]: Department of Physics, University of Ulsan, Ulsan 44610, Korea.

Contents

1. **Sum rule calculation for orbital to spin magnetic moment ratio**
2. **Estimation of the in-plane *d*-spacings of Co layers in the superlattices**
3. **First-principles calculation**
   3.1 Method
   3.2 Comparison between partial DOS of *d* orbitals with strained and unstrained interfaces
   3.3 Analyses of Co magnetic moments in the un-strained and the strained Co/Pd
4. **Hydrogenation effect on the MCA energy**
5. **Determining of magnetic anisotropy energy by the area method**

1. Sum rule to calculate the ratio of orbital to spin magnetic moment

The x-ray magnetic circular dichroism (XMCD) was measured with 95% circularly polarized incident light in the normal direction of the superlattice plane at the 2A beamline of the Pohang Light Source. The total electron yield mode was used to obtain spectra at 300 K. An XAS measurement was performed at the Co L-edge to extract the XMCD spectra. XAS spectra from $2p \rightarrow 3d$ dipole transitions represent the dependence on the parallel (μ_+) and anti-parallel alignments (μ_-) of the magnetization directions with the photon helicity, as shown in **Figure. S1**.

According to the sum rule, the m_o/m_s ratio can be quantitatively obtained as

$$\frac{m_0}{m_s} = \frac{2\int_{L_3+L_2}(\mu_+ - \mu_-)dE}{9\int_{L_3}(\mu_+ - \mu_-)dE - 6\int_{L_3+L_2}(\mu_+ - \mu_-)dE} = \frac{2q}{9p - 6q}, \quad (1)$$

where p and q are negative in this case, which means that the directions of the orbital and spin magnetic moments in the superlattices are parallel. The m_o/m_s ratio is an important parameter when investigating how the orbital moment contributes to spin-orbit interaction in the superlattices.[1,2]

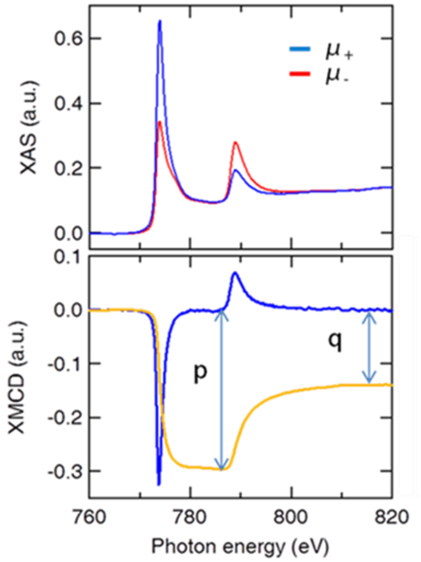

Figure S1. XAS and XMCD spectra of the [M-Co/Pd] with a 4-Å-thick Co layer measured at the Co $L_{2,3}$ edges. The red and blue lines are XAS spectra measured when magnetizations of the superlattice were anti-parallel and parallel, respectively, to the incident direction of the 95% circular polarized x-ray. The solid yellow line is the integrated XMCD spectrum. p and q are explained in the article.

2. Estimation of the in-plane *d*-spacings of Co in the superlattices

A noticeable feature in the grazing incident x-ray diffraction (GIXRD) spectra of the [M-Co/Pd] and the [R-Co/Pd], as shown in **Figures S2 (a)** and **(b)**, is that the shoulders of the primary peak, which are diffracted from Co (220) planes, start to be observed at t_{Co}=~6Å and increase in their intensity with the t_{Co} for both [M-Co/Pd] and [R-Co/Pd], which has also been reported in the literature.[3,4] **Figure S2 (c)** shows a schematic of the measurement setup. The primary peaks correspond to the (220) planes of the superlattice films whose normal vector is parallel to the in-plane direction. The primary peaks are resolved into three peaks (dotted lines **in Figures. S2 (a) and (b)**) based on the following two observations: 1) The intensity of the peak at the low angle in the primary peak increases relative to that at high angles when α is decreased from 0.33° to 0.22°. Because surface scattering can be enhanced at low incident angles, the peak at the low angle can mainly originate from the top of the film, that is, the Pd capping layer. 2) The intensity of the peak at the high angles does change considerably with the t_{Co}, unlike the peak at the low angle, which implies that the peaks at the high angles can be assigned to the rest of the superlattice. **Figures S2 (d)** shows the anomalous x-ray scattering (AXS) spectra of the superlattices near the Co *K*-edge measured at the primary (220) Bragg peak in the GIXRD spectra. The variations of the integrated intensities as a function of the x-ray photon energy show intensity cusps near the Co *K*-edge for the selected thicknesses of the Co layer in the superlattices, which proves that the Co layers in the superlattices contribute to the primary (220) Bragg peak. The interatomic distances between Co atoms along the in-plane direction are estimated using the interplanar spacings (*d*-spacings) of (220) for both strained and relaxed Co in terms of a compositional average *d*-spacing, as discussed below. **Figure S3** shows the *d*-spacings obtained by the curve-fitting of the GIXRD spectra as a function of the t_{Co}. On the other hand, an x-ray absorption spectroscopy (XAS) study confirms that the Co_3O_4 superlattices are mostly reduced to the metallic phase. Therefore, all effects of the residual oxide phases in the [R-Co/Pd] are neglected in this study.

According to our observation, the strain state of both superlattices can be divided into three regimes in terms of the t_{Co}, as indicated in **Figure S3.** The coherent regime (denoted as Regime I) is defined in the t_{Co} range of 0–5 Å, where the strain was coherently sustained. When the t_{Co} range is 6–9 Å, the intermediate regime (Regime II) is defined, where an abrupt decrease in the $d_{Co(220)}$ was observed; and the incoherent regime (Regime III) is defined when t_{Co}>9 Å, where $d_{Co(220)}$ is similar to that of the [M-Co/Pd]. The GIXRD results show that the [R-Co/Pd] have $d_{Co/Pd\,(220)}$ larger than that of the [M-Co/Pd] by ~0.73% throughout the entire thickness range of Co. In other words, the *d*-spacings of the [R-Co/Pd] are closer to those of bulk Pd than the *d*-spacings of the [M-Co/Pd] are. The drop in $d_{Co(220)}$ for the [R-

Co/Pd] in Regime II starts at the t_{Co} thicker than the t_{Co} for the [M-Co/Pd], which implies that the Co layers in the [R-Co/Pd] was under an in-plane tensile stress much larger than the stresses in the [M-Co/Pd]. The $d_{Co(220)}$s of both superlattices become close to each other in Regime III because their tensile stress in the strain-relaxed Co layer along the in-plane direction becomes comparable. In Regimes II and III, the volume-weighted average d-spacing (d_{ave}) was used for the precise estimation of the magnetoelastic anisotropy because the Co layers have d-spacings from both a strained and a strain-relaxed lattice.

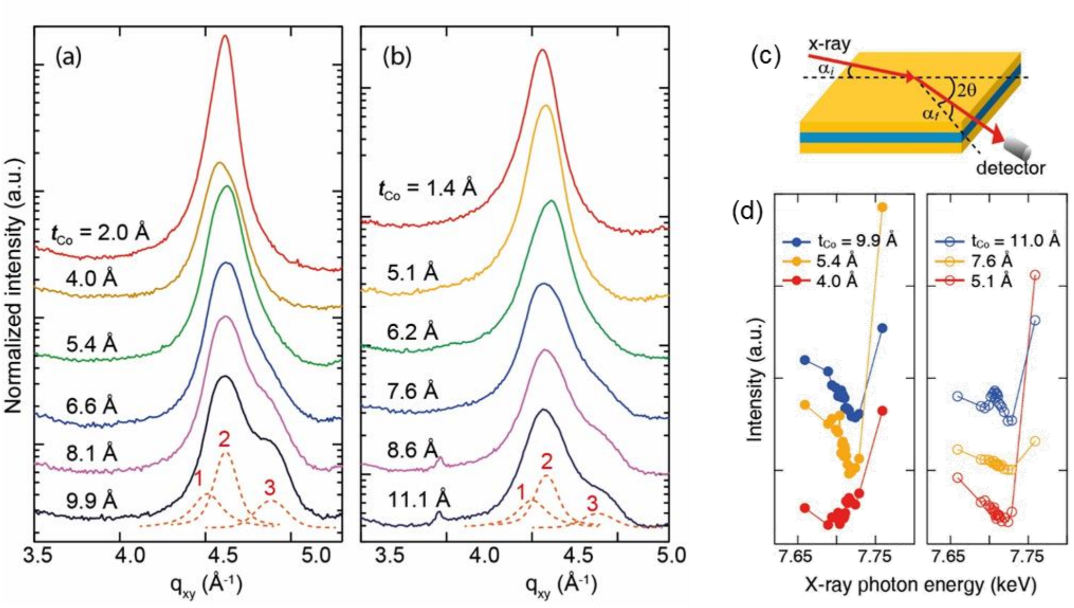

Figure S2. In-plane GIXRD spectra of (a) the [M-Co/Pd] and (b) the [R-Co/Pd]. Three peaks are considered for curve-fitting of the primary (220) peak: 1. Pd capping layers, 2. strained Co and Pd layers, and 3. relaxed Co layers. (c) A schematic of the GIXRD measurement setup. α_i and α_f are the incident and reflection angles of the x-ray. (d) Absorption x-ray spectra of the [M-Co/Pd] (left) and the [R-Co/Pd] (right) at the diffraction angle of the primary peak.

The following three assumptions are made to estimate d_{ave}: 1) The Co layers are pseudomorphically grown on the Pd layers at the interfaces, while the Co lattices in the middle of Co layer have $d_{Co(220)}$ close to the bulk value due to the strain relaxation. 2) Because the gradual decrease in the $d_{Co(220)}$ with t_{Co} was observed in the experiments, we assume that the superlattice system has a strain gradient[5] out of plane. And 3) The d_{ave} can be estimated using Equation (3) in our case:

$$d_{Co}^{ave} = \frac{\left[0.5 \cdot \left(d_{Co}^{rel} + d_{Co}^{str}\right) \cdot n_{Co}^{str} + d_{Co}^{rel} \cdot n_{Co}^{rel}\right]}{n_{Co}}. \tag{S3}$$

Here $n_{Co}^{rel} = n_{Co} - n_{Co}^{str}$, where n_{Co}^{str} and n_{Co}^{rel} are the numbers of Co monolayers (MLs) with pseudomorphism and strain relaxation, respectively. The first term in the square bracket in Equation (S3) indicates the total thickness of Co before relaxation or the sum of the *d*-spacings with a gradual decrease in the $d_{Co(220)}$, whereas the second term represents the total thickness of Co after relaxation. The estimated d_{ave} from the GIXRD results are shown in **Figure S3 (bottom)**.

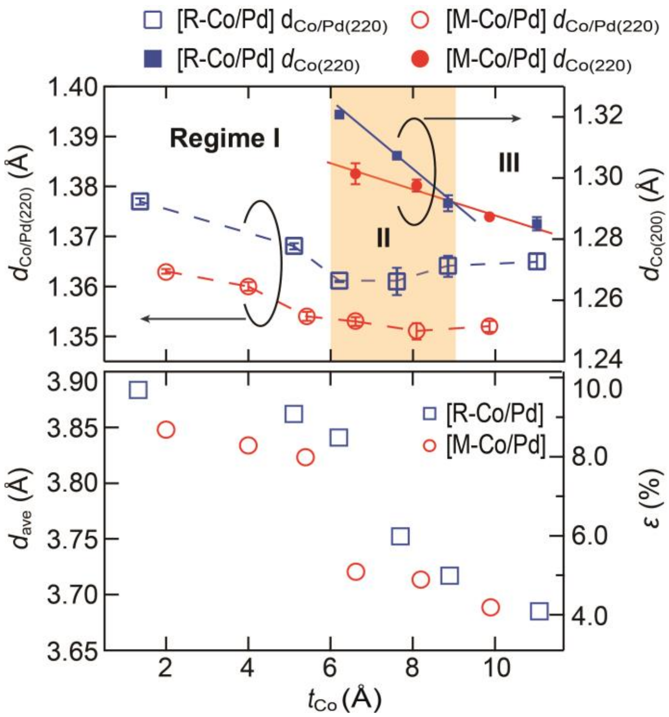

Figure S3. (top) The *d*-spacings of the (220) of the Co layers and the Co/Pd superlattices in the [M-Co/Pd] and the [R-Co/Pd]. (bottom) Calculated d_{ave} and ε.

3. First-principles calculations

3.1 Method

First-principles density functional theory calculations were performed using the Vienna *ab-initio* simulation package[6] with the generalized gradient approximation[7] for the exchange-correlation potential. To reveal the origin of the enhanced PMA of the reduced Co_3O_4/Pd superlattice observed in the experiment, we investigated superlattices composed of 4-ML Pd and 5-ML Co, whose geometry is shown in **Figure S4**. To consider the 9.8% mismatch between the two-dimensional (2D) lattice constants of bulk Co (3.54 Å) and Pd (3.89 Å), we employed 2D lattice strains of 0% to 10%. Here, 0% strain implies the 2D lattice constant of Co (3.54 Å), and 9.8% strain corresponds to Pd (3.89 Å). Furthermore, we considered the 5-ML Co thin films without Pd. This was to reveal the Pd interface effect more explicitly by comparing the results of the Co thin films and the superlattices. The *k* meshes in Brillouin-zone sampling were 18×18×2 and 18×18×1 for the Co/Pd superlattice and the Co thin film, respectively. The magnetocrystalline anisotropy (MCA) energy (E_{MCA}) is defined here as the difference between the total energies of the in-plane and the out-of-plane and magnetization directions; i.e., $E_{MCA}=E_{\rightarrow}-E_{\uparrow}$. In our simulation, the strained Co/Pd corresponds to the [R-Co/Pd] in the article.

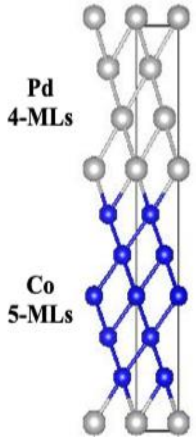

Figure S4. Co/Pd superlattice: 4-ML Pd on top of 5-ML Co.

3.2 Comparison between partial DOS of *d* orbitals with strained and unstrained interfaces

Figures S5(a) and (b) show the partial density of states (PDOS) of the *d* orbitals, decomposed into the d_{xy,x^2-y^2}, $d_{yz,xz}$, and d_{z^2} orbitals, which correspond to the magnetic quantum numbers m=±2, m=±1, and m=0, respectively. To confirm the interface and surface effects on the magnetocrystalline anisotropy (MCA) energy, which reflects the orbital anisotropy, we separately present Pd (I) and Co (I) of the superlattice and Co (S) and Co (S-1) of the Co film, where *I* and *S* denote the interface and surface, respectively, and (S-1) represents the subsurface, one layer below the surface. Compared with the unstrained Co/Pd superlattice, the occupied majority m=±1 orbitals, and the unoccupied minority m=0 orbitals of Co (I) and Pd (I) shift toward the Fermi level under 9% strain (arrows in **Figure S5 (d)**). As a result, $\langle m=\pm1,\uparrow|L_x|m=0,\downarrow\rangle$ becomes stronger, which leads to the significantly enhanced PMA for the strained Co/Pd superlattice. Here, the up-spins (↑) and down-spins (↓) indicate the majority and minority spins, respectively. Similar behaviors are observed for the occupied majority m=±1 orbitals and the unoccupied minority m=0 orbitals of Co (S), which are denoted by arrows in **Figure S5(d)**, as previously mentioned in the discussion on the MCA of the Co/Pd superlattice. However, a prominent peak of the minority m=±1 orbitals appears near the Fermi level of Co (S) under 9.8% strain (dotted arrow in **Figure S5(d)**), which negatively contributes to the MCA through $\langle m=\pm1,\uparrow|L_x|m=0,\downarrow\rangle$ at the surface. This negative contribution is balanced by the positive contribution of $\langle m=\pm1,\uparrow|L_x|m=0,\downarrow\rangle$.

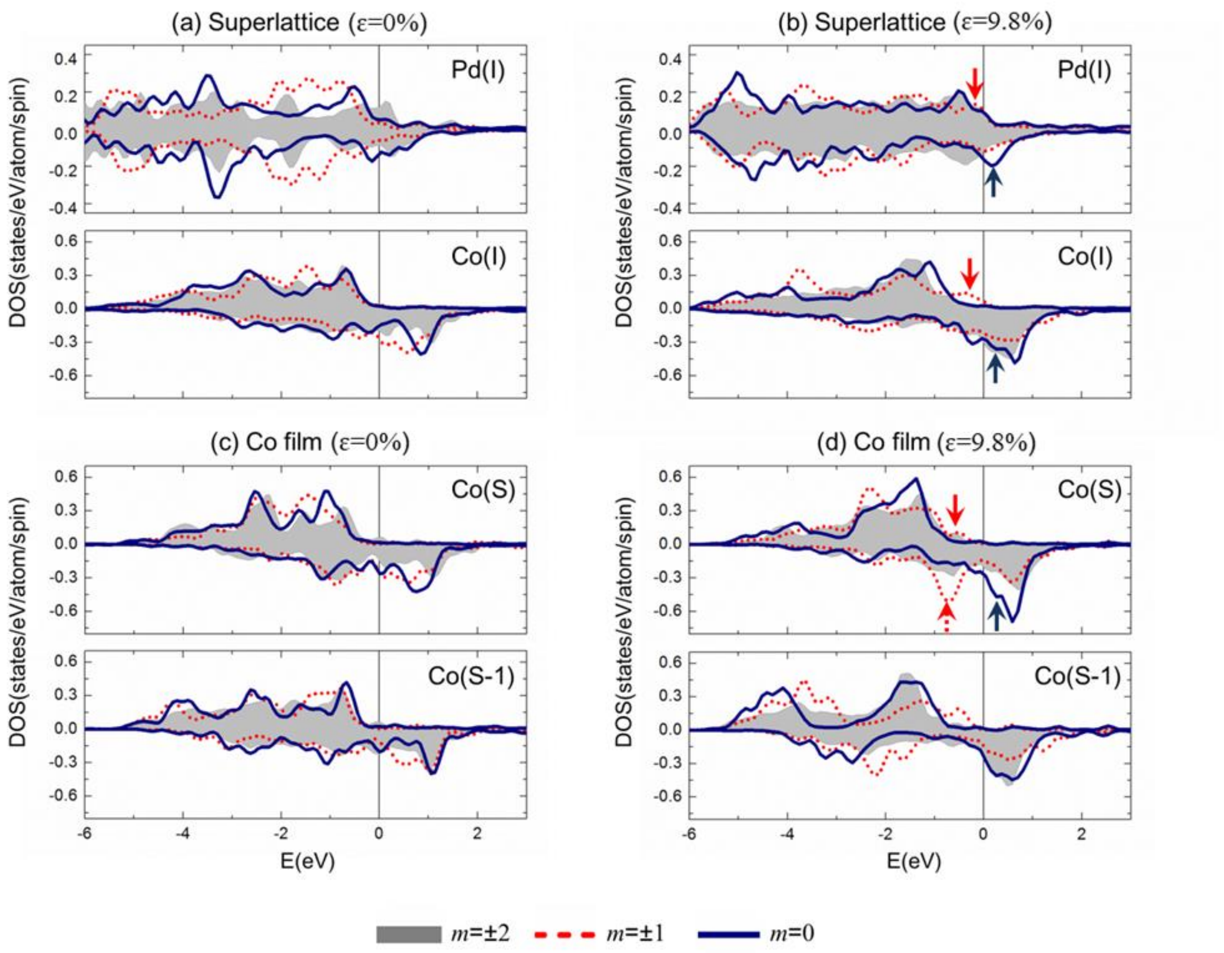

Figure S5. Partial DOS of *d* orbitals of (a) the unstrained superlattice, (b) the superlattice with 9.8% strain, (c) the unstrained Co film, and (d) the Co film with 9.8% strain. I, S, and S-1 denote the interface, surface, and subsurface, respectively. The shaded region and the dashed and solid lines indicate m=±2, m=±1, and m=0, respectively. The Fermi level is set to zero in energy.

3.3 Analyses of Co magnetic moments in the un-strained and the strained Co/Pd

Table S1. Comparison of calculated total magnetic moments Co of the un-strained and strained Co/Pd

superlattice	Unstrained Co/Pd		Strained Co/Pd	
	$m^{\perp}$	$m^{\parallel}$	$m^{\perp}$	$m^{\parallel}$
m_{tot} (μ_B)	8.78	8.73	10.12	9.92
$\Delta m = m^{\perp} - m^{\parallel}$ (μ_B)	0.04		0.20	
m_o (μ_B)	0.425	0.409	0.722	0.573
$\Delta m_o = m_o^{\perp} - m_o^{\parallel}$ (μ_B)	0.016		0.149	
m_o/m_s	0.622	0.572	0.919	0.784

Table S2. Orbital moments of Co at the interface in the un-strained and the strained Co/Pd

Co at interface	[M-Co/Pd]		[R-Co/Pd]	
	$m^{\perp}$	$m^{\parallel}$	$m^{\perp}$	$m^{\parallel}$
m_o (μ_B)	0.078	0.070	0.124	0.092
$\Delta m_o = m_o^{\perp} - m_o^{\parallel}$ (μ_B)	0.008		0.032	
m_o/m_s	0.047	0.039	0.070	0.052

The analyzed orbital moments of Co in the un-strained and the strained Co/Pd and at the interface of Co/Pd are listed in Tables S1 and Table S2. The perpendicular components of total magnetic moment of Co in both Co/Pd are larger than the in-plane components, and the difference between the components is denoted Δm. The change in total orbital moments (Δm_o), which is a measure of orbital anisotropy, is 0.016 and 0.149 μ_B for the un-strained and the strained Co/Pd, respectively. The m_o/m_s is also estimated for each case.

In Table S2, the orbital moments of Co *only* at the interface are listed. The Δm_o are 0.008 and 0.032 μ_B for the un-strained and the strained Co/Pd, respectively. We note here that the magnitudes of the magnetic moments in Table S2 are much smaller than those in Table S1 simply because the orbital moments of all Co in the superlattice are calculated and listed in Table S1. We confirmed that the magnetic moment of Co at the interface is the largest. Though the orbital moment from the sub-interface contribution is not negligible, the orbital moment from the interface contribution is sufficiently large to demonstrate the main argument of this article.

4. Hydrogenation effect on MCA energy

To investigate the hydrogen effect on the MCA energy (E_{MCA}), we considered six different hydrogen sites, which were positioned at the tetrahedral and octahedral sites in the Co, Pd, and Co-Pd interface layers. Tetrahedral sites are energetically more stable than octahedral sites for all atomic layers. The hydrogen atom in the Co layer energetically favors over that in the Pd layer and the Co-Pd interface by 0.93 and 0.72 eV/f.u., respectively. As shown in Figure S6a, hydrogen contributes in-plane MCA regardless of its location, which is consistent with previous results.[8] The hydrogen atom in the Co layer significantly reduces the MCA energy.

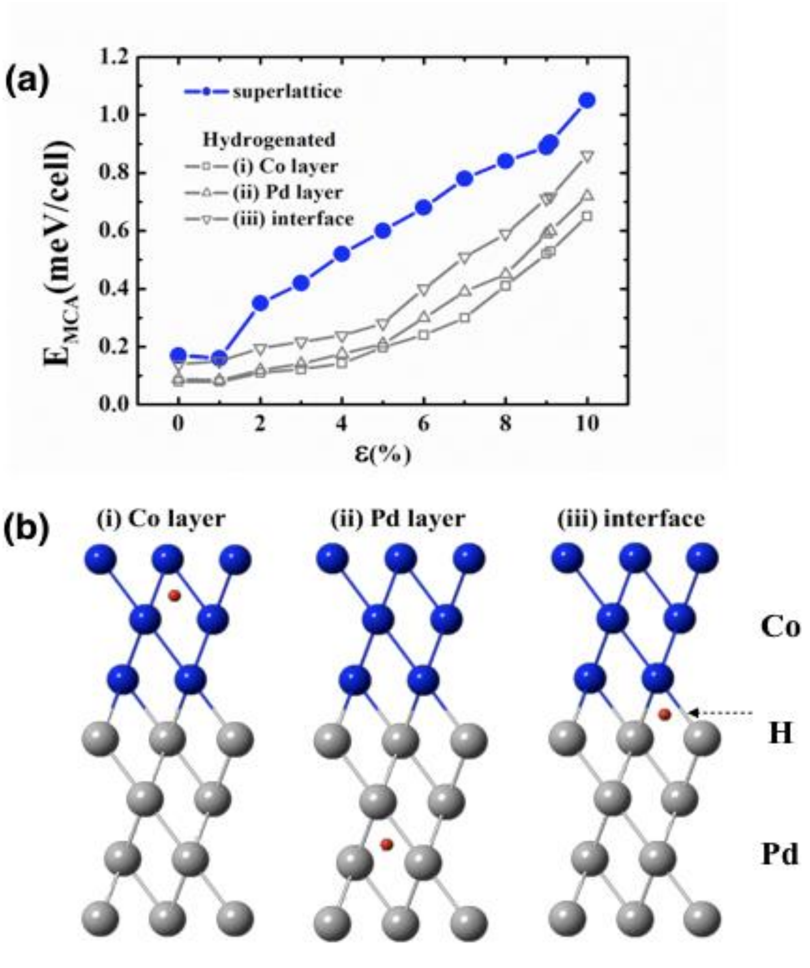

Figure S6. (a) E_{MCA} as a function of the strain and (b) three different interstitial sites of hydrogen in the Co/Pd superlattice for calculations. Blue, grey, and red spheres denote Co, Pd, and H, respectively.

5. Magnetic anisotropy estimation by areal method

The magnetic anisotropy energies (Ku) were estimated by the areal method [9]. At first, we measured the magnetic hysteresis loops for Co/Pd superlattices with a field-sweep along the in-plane (or the hard axis) and the film-normal (or the easy axis) directions using a vibrating sample magnetometer (VSM). Then, we estimated the magnetic anisotropy energies by calculating the difference in the area of the first quadrant of a hysteresis loop (H > 0; M > 0) between the two loops, the easy and the hard axis loops. The difference in the area for typical [R-Co/Pd] is shown as a shaded region in Fig. S7.

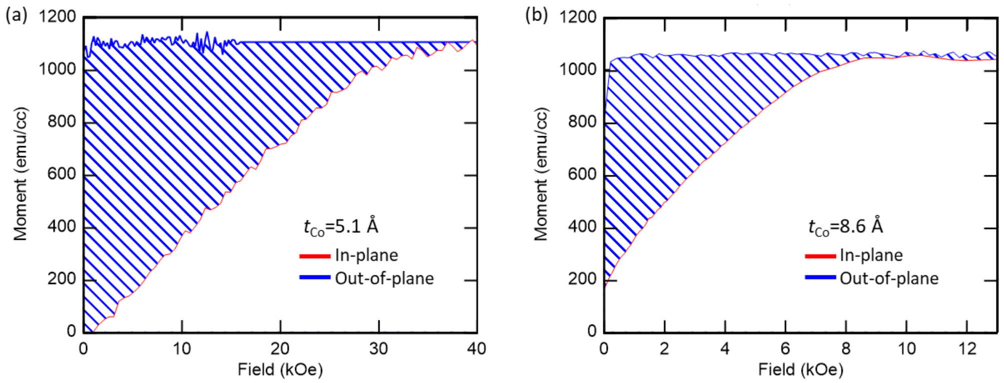

Figure S7. The first quadrant of a hysteresis loop (H > 0; M > 0) of [R-Co/Pd] with t_{Co}= (a) 5.1 Å and (b) 8.6 Å under the out-of-plane and the in-plane field sweeping.

REFERENCES

[1] C. T. Chen, Y. U. Idzerda, H.-J. Lin, N. V. Smith, G. Meigs, E. Chaban, G. H. Ho, E. Pellegrin, F. Sette, *Phys. Rev. Lett.* **1995**, *75*, 152.

[2] P. Gambardella, S. Rusponi, M. Veronese, S. S. Dhesi, C. Grazioli, A. Dallmeyer, I. Cabria, R. Zeller, P. H. Dederichs, K. Kern, C. Carbone, H. Brune, *Science* **2003**, *300*, 1130.

[3] P. Bruno, J.-P. Renard, *Appl. Phys. A* **1989**, *49*, 499.

[4] M. T. Johnson, P. J. H. Bloemen, F. J. A. den Broeder, J. J. de Vries, *Rep. Prog. Phys.* **1996**, *59*, 1409.

[5] T. Kingetsu, K. Sakai, *Phys. Rev. B* **1993**, *48*, 4140.

[6] G. Kresse, J. Furthmüller, *Phys. Rev. B* **1996**, *54*, 11169.

[7] J. P. Perdew, K. Burke, M. Ernzerhof, *Phys. Rev. Lett.* **1996**, *77*, 3865.

[8] S. Kim, S. Lee, J. Hong, *ACS Nano* **2014**, *8*, 4698.

[9] M. T. Johnson, P. J. H. Bloemen, F. J. A. den Broeder, J. J. de Vries, *Rep. Prog. Phys.* **1996**, *59*, 1409.
